# Coordination between Eye Movement and Whisking in Head-Fixed Mice Navigating a Plus Maze

**DOI:** 10.1523/ENEURO.0089-22.2022

**Published:** 2022-08-26

**Authors:** Ronny Bergmann, Keisuke Sehara, Sina E. Dominiak, Jens Kremkow, Matthew E. Larkum, Robert N. S. Sachdev

**Affiliations:** 1 NeuroCure Cluster of Excellence, Charité–Universitätsmedizin Berlin, D-10117 Berlin, Germany; 2 Institute of Biology, Humboldt Universität zu Berlin, D-10117 Berlin, Germany; 3 Charité–Universitätsmedizin Berlin, D-10117 Berlin, Germany; 4 Bernstein Center for Computational Neuroscience Berlin, D-10115 Berlin, Germany

**Keywords:** sensory motor systems, visual tactile coordination, orienting behavior, movement prediction, vibrissae

## Abstract

Navigation through complex environments requires motor planning, motor preparation, and the coordination between multiple sensory–motor modalities. For example, the stepping motion when we walk is coordinated with motion of the torso, arms, head, and eyes. In rodents, movement of the animal through the environment is coordinated with whisking. Even head-fixed mice navigating a plus maze position their whiskers asymmetrically with the bilateral asymmetry signifying the upcoming turn direction. Here we report that, in addition to moving their whiskers, on every trial mice also move their eyes conjugately in the direction of the upcoming turn. Not only do mice move their eyes, but they coordinate saccadic eye movement with the asymmetric positioning of the whiskers. Our analysis shows that asymmetric positioning of whiskers predicted the turn direction that mice will make at an earlier stage than eye movement. Consistent with these results, our observations also revealed that whisker asymmetry increases before saccadic eye movement. Importantly, this work shows that when rodents plan for active behavior, their motor plans can involve both eye and whisker movement. We conclude that, when mice are engaged in and moving through complex real-world environments, their behavioral state can be read out in the movement of both their whiskers and eyes.

## Significance Statement

Natural behavior is multimodal and occurs in multiple sensory motor dimensions. In rodents, whisker use can reflect the behavioral state of the animal (i.e., whether mice are walking, running, standing still, or turning). Here we show that while navigating a plus maze, head-fixed mice coordinate the movement of their eyes and whiskers. While eye and whisker movement both predict the turn direction mice are about to take as they exit and enter lanes, whisker positioning predicts turn direction earlier than eye movement does. These results reveal aspects of the multimodal coordination among visual, somatosensory, and motor systems that guides behavior in mice.

## Introduction

One of the fundamental activities of the brain is to monitor and control the movement of the body. A simple everyday act like walking is associated with a sequence of movements that involve the body, limbs, head, and eyes (Bizzi et al., 1971; Grasso et al., 1998; Dietz et al., 2001; Dietz, 2002; Land, 2006; Foulsham, 2015). Despite the obvious importance of understanding the coordination between various sensory–motor elements engaged in planning and coordinating behaviors, the details of this coordination are still not completely clear.

In rodents, exploration of the environment is often linked to movement of the head and whiskers (Hartmann et al., 2003; Towal and Hartmann, 2006; Arkley et al., 2014; Sofroniew et al., 2014), with facial movements linked to activity observed in widespread areas of cortex (Musall et al., 2019; Steinmetz et al., 2019; Stringer et al., 2019). Eye movements, which are thought to have “just in time” and “look-ahead” function in many species (Land and Hayhoe, 2001; Land, 2006; Mennie et al., 2007; Land, 2009; Foulsham, 2015; Srivastava et al., 2018), have often been overlooked in rodents. Part of the reason for this neglect is related to the difficulty in measuring the motion of the small rodent eyes, especially in freely moving animals (Payne and Raymond, 2017; Meyer et al., 2018, 2020). Additionally, rats and mice are nocturnal animals with a highly developed somatosensory system (Woolsey and van der Loos, 1970), where the relationship between whisking and navigation is observable more easily (Hartmann et al., 2003; Towal and Hartmann, 2006; Arkley et al., 2014; Sofroniew et al., 2014; Dominiak et al., 2019). But in the last decade there has been an evolution in our thinking as it has become increasingly obvious that in addition to using their tactile system, rodents can also use their visual system to guide their response to predators, and can and do move their eyes to guide navigation (Wallace et al., 2013; Yilmaz and Meister, 2013; Hoy et al., 2016; Meyer et al., 2020). Together, it is likely that even in rodents, eye movement is coordinated with actions in other sensory motor dimensions (i.e., for motor planning and for coordinating movement during navigation; Land, 2009).

Earlier work has shown that when mice plan their movement in a real-world floating maze, they begin to position their whiskers asymmetrically; the asymmetry reflects the direction that mice turn in as they navigate the maze (Dominiak et al., 2019). A key limitation of this earlier work, and of work in the rodent motor systems in general, has been our fixation on the highly specialized vibrissae system and whisking. The earlier work has not been clear about whether and how much mice coordinate the movement of their body with movement of both their eyes and whiskers. Furthermore, whether changes in the position of whiskers or eyes indeed reflect motor planning has not been examined, because it has not been feasible. Here we extended the work of Dominiak et al. (2019) by training mice to overcome their natural handedness preference and monitored both whisker and eye movement bilaterally. Our work shows that whisking is coordinated with the movement of the eyes, that mice move their eyes in a “look-ahead” fashion, and that both eye movement and whisking are related to turn direction.

## Materials and Methods

### Behavioral experiments

We performed all procedures in accordance with protocols for the care and use of laboratory animal approved by Charité–Universitätsmedizin Berlin and Berlin Landesamt für Gesundheit und Soziales (LaGeSo).

#### Surgery

Adult C57BL/6 male mice (*n* = 4), weighing 25–32 g were anesthetized with ketamine/xylazine (90 mg/kg/10 mg/kg). Lightweight aluminum head posts were affixed to the skull using Rely X and Jet Acrylic (Ortho-Jet) black cement (Dominiak et al., 2019; Ebner et al., 2019). In the 2 d after surgery, analgesia was provided by buprenorphine and carprofen injections.

#### Airtrack

The Airtrack consists of the following three parts: (1) a square Plexiglas airtable with tiny, evenly spaced holes for holding the platform, a circular maze aloft; (2) a lightweight, circular maze that floats on the bed of air created; and (3) a pixy camera that tracks the position of the maze (Nashaat et al., 2016; Dominiak et al., 2019). Each hole of the airtable has a plastic ball bearing that moved to the mouth of the hole preventing airflow when the platform was not covering that particular hole. This reduced the hissing sound associated with the outflow of pressurized air. For the floating maze, Dominiak et al. (2019) used a platform that weighed 160 g and was 30 cm in diameter. In this study, we used a Styrofoam base with a milled out Plexiglas ring on the borders that reduced friction with the walls of the airtable. Our plus maze had a diameter of 22 cm and weighed 30 g. It had four 8-cm-long, 2.5-cm-high identical opaque (black or red Plexiglas) lanes, each with the same texture engraved on the walls. One end of all lanes, toward the center of the maze, opened into a circular area, in which mice could turn and exit or enter lanes. The other end of the lane opened toward a lick port, which was attached to a linear actuator. A pair of LEDs, 7 cm apart, positioned directly in front of each eye of the mice, were set up on each side of the lick port and were used to indicate whether the trial was a right or left turn trial.

When mice were head fixed, their head post jutted over the walls of the lanes, but the head of the mouse was below the top of the wall. External visual cues were minimized by covering the setup with a black cloth. Note, however, that the black/UV light (365–400 nm) used for tracking and fluorescing whiskers illuminated the setup.

The pixy camera positioned under the platform used a color code (Nashaat et al., 2016) to track the position, the direction of movement, and the speed of movement of the maze as the head-fixed mouse guided it along. When mice entered the correct lane, the pixy output was used to trigger a motor that lowered the lick port into position.

#### Plus maze task

**Figure 1.**
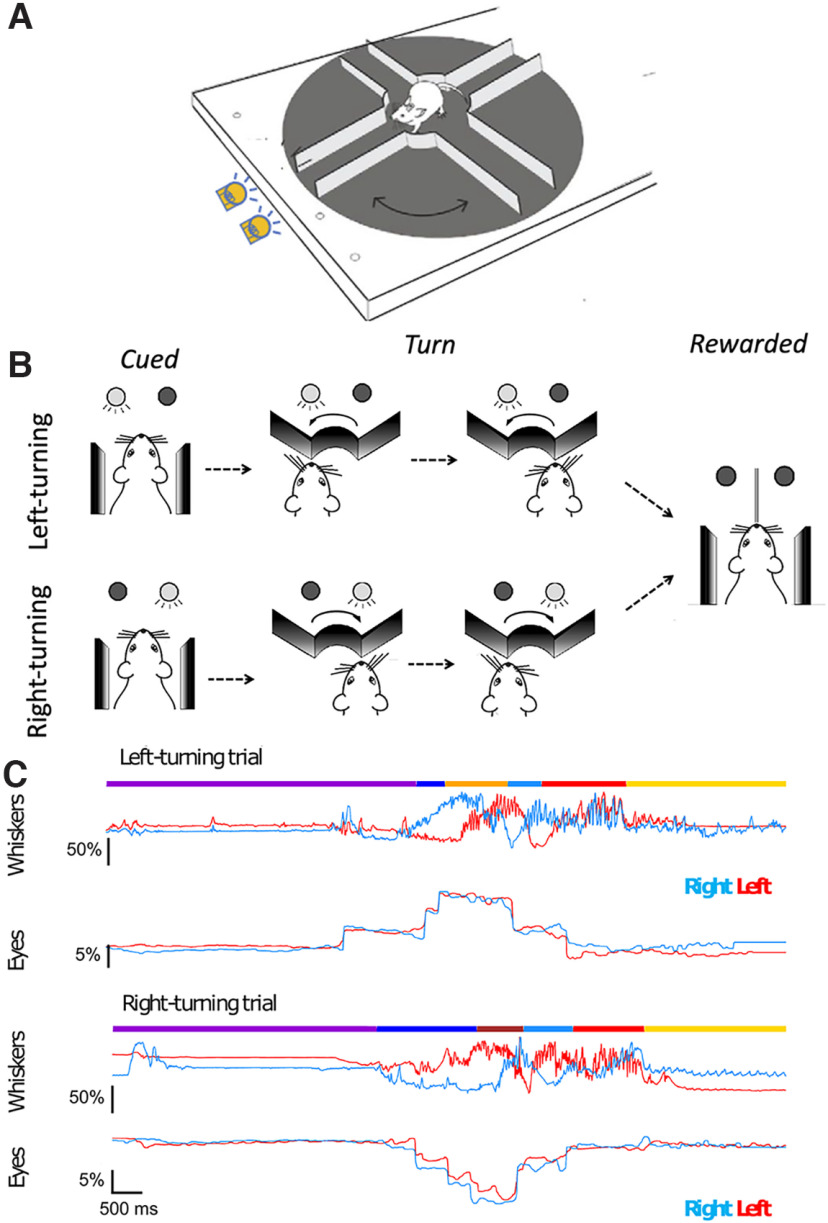
Schematic of trials, and behavioral state estimates during whisker and eye movements. ***A***, ***B***, Schematic of the task. A trial starts with the animal at the end of the last rewarded lane. One of the two cue LEDs indicate the direction the animal should take next. The right LED indicates that for the trial mice had to move in a clockwise “right turn” direction and enter the adjacent lane where they wait for the reward tube to descend. The left LED indicated a left turn trial. ***C***, Single right-turn and left-turn trials, showing whisker and eye movement. Mice move backward at their own pace, reach the center of the maze, and turn left (top) or right (bottom) until the reward LED turns off signaling that the mouse was at the mouth of the correct, rewarded lane. Mice learn to expect a reward at the end of the lane. Once the lick spout was in their reach, mice lick the reward. During this behavior, whiskers on two sides of the face (left-side whiskers, blue; right side whiskers, red) move in opposite directions for left (top) and right (bottom) turns. The side-to-side asymmetry becomes evident just as, or just before, mice begin to move backward in the lane. By comparison, the eyes (right eye, blue trace; left eye, red trace) move in a conjugate fashion on both sides of the face. The behavioral state was manually annotated: purple, end of lane; dark blue, backward movement; brown, right turns; orange, left turns; light blue, forward; red, expect reward; yellow, lick. Scale bars represent the amplitude of movement relative to the full amplitude of whisker motion (for whiskers) or relative to the width of the eye (for eyes) during the session.

Mice were trained to use LED cues to turn the maze in the cued direction (Fig. 1). A trial began when mice finished with the reward from the previous trial and were still at the end of a lane. One of two LEDs turned on, indicating the expected turn direction for the ensuing trial (Fig. 1*A*,*B*, Movie 1; generated using real time tracking of pupils). The light was left on until mice had propelled themselves backward, out of the lane, and had begun to turn in the expected direction. When they were at the entrance to the correct adjacent lane, the LED was switched off. Note that there are no time constraints on the mice: they could move as fast or as slowly as they wanted, and they could make turns as many times as they wanted. Once a trial ended, mice could start the trial when they wanted and could go as fast or as slow as they wanted to.

Movie 1. Real-time eye tracking and behavior on two trials. A machine-learning algorithm was used to track eye position in real time and to illustrate how eye position changed with the position of the mouse in the maze. 10.1523/ENEURO.0089-22.2022.video.1

#### Training

One week after surgery, mice were habituated to being handled, and to the Airtrack plus maze platform. In the first days of habituation, mice acclimated to having their head post handled by the experimenter and to short (up to several minutes) head fixation. In the course of the first week of habituation, the duration of head fixation was gradually increased from 5 to 40 min. In addition to head fixation, mice were habituated to having their whiskers painted. During whisker painting, mice were rewarded with sweetened condensed milk.

After a week of habituation, mice were water deprived and were trained to move the platform around themselves. At the beginning of this training phase, the experimenter manually nudged or guided the animal. Over the course of 14 consecutive days of training, mice learned to move the maze by themselves, and gradually increased the number of trials they performed. Note that there were no temporal constraints on mice; the trials were self-initiated, and each behavioral epoch and each trial could be as long or as short as mice made them.

#### Data acquisition

Video data from painted whiskers and the movement of the eyes were collected as mice performed the task (Fig. 1*C*). We manually annotated 113 trials taken from 21 sessions in four animals. These 113 trials were used to examine the effect of the behavioral states of the animal on whisker positions. To examine the relationship between behavioral states and eye positions, we visually validated the alignment between behavioral (whisker) videos and eye videos. After excluding the trials where behavioral and eye videos did not align properly with each other, we used 91 trials from 17 sessions recorded in four animals for the analysis of eye positions (Table 1).

**Table 1:** Summary of sample sizes

Animal ID	Session	Trials Anno+Whis	Trials Anno+ Whis+Eyes
MLA005756	#1	6	6
	#2	8	8
	#3	7	7
	#4	6	6
	#5	6	6
MLA005757	#1	6	6
	#2	6	6
	#3	5	5
	#4	6	6
	#5	5	2
	#6	6	6
MLA007518	#1	6	6
	#2	6	6
	#3	6	6
	#4	5	0
	#5	4	2
MLA007519	#1	3	0
	#2	3	3
	#3	5	0
	#4	4	0
	#5	4	4
Trials in total		113	91
Sessions in total		21	17
Animals in total		4	4

The numbers of trials with manual annotation of states (anno), with whisker tracking (whis) and with valid eye tracking (eyes) are shown. Because of validation after alignment of eye videos with behavior videos, the number of trials with eye tracking is smaller than the other types of trials.

To track the behavioral states and whisker positions, behavioral data were recorded at a resolution of 1200 pixels in width × 700 pixels in height at 200 Hz with a camera (acA1920-155uc USB 3.0, Basler) and an *f* = 25 mm/F1.4 objective being set above the animal. The C2 whiskers on both sides were painted green (UV glow; https://www.uvglow.co.uk/), and illuminated by UV torches. Our goal was to capture whisking movement, which in mice can go up to ∼20 Hz (Jin et al., 2004; Mitchinson et al., 2011; Sofroniew et al., 2014; Dominiak et al., 2019). A frame rate of 200 Hz is well above the Nyquist frequency and is sufficient for this. Note that our work follows on the work by Dominiak et al. (2019), which showed that tracking a single whisker on each side of the face was sufficient to determine both the setpoint and amplitude of whisker movement and that the tactile events, the very high-frequency slip stick events, do not explain the behaviors that are at the core of this work. Mice perform the behavior and move their whiskers in a similar manner after whiskers have been trimmed off.

Videos were acquired in the proprietary format from Matrox Imaging (https://www.matrox.com/) and later converted into the H.264 format. The acquisition and conversion were accomplished using ZR-view, a custom software (Robert Zollner, Eichenau, Germany).

For recording the pupils of the animal, two cameras (acA1300-200um USB 3.0, Basler), each with an *f* = 50 mm/F2.8 objective, were used. Two independent infrared light sources (model LIU850A, Thorlabs; https://www.thorlabs.com/) were directed toward the eyes. Video files of both pupils were acquired at a resolution of 192 pixels in width × 200 pixels in height at 100 Hz using Pylon-PD (a custom software made by Eridian Systems, Berlin, Germany).

An Atmel ATmega328P microcontroller was reprogrammed to generate synchronized 100 and 200 Hz trigger pulses for simultaneous frame acquisition from the three cameras (i.e., two pupil cameras and the behavioral camera). The Mega board (Arduino) that monitored Airtrack movement was also used to control the trial-based data acquisition. Trials were separated by two consecutive transistor–transistor logic (TTL) level pulses; the first TTL marked the end of a trial and the second one marked the start of a new trial. These TTL signals were used to trigger the start and the end of video acquisition.

Behavioral states in each trial were annotated manually using the behavioral video files, by marking the frames when state transitions occurred (Fig. 1*C*), as follows: quietly sitting at end-of-lane, moving backward, turning, moving forward, waiting for reward, or licking. Entry into or exit from a lane were annotated by using the position of the nose in relation to the edges of the lanes. The frame on which the animal started moving continuously in one direction was defined as the onset of forward or backward movement.

### Data analysis

#### Data selection and analysis

The 91 trials taken from 17 sessions in four animals accounted for 728 annotated behavioral epochs that were used for data analysis. The following analytical procedures were performed using Python (version 3.7.6; https://www.python.org/), along with several standard modules for scientific data analysis [NumPy, version 1.18.1 (https://www.numpy.org/); SciPy, version 1.4.1 (https://www.scipy.org/); matplotlib, version 3.1.3 (https://matplotlib.org/); pandas, version 1.0.1 (https://pandas.pydata.org/); scikit-learn, version 0.22.1 (https://scikit-learn.org/stable/); Bottleneck, version 1.3.2 (https://pypi.org/project/Bottleneck/); Statsmodels, version 0.13.0 (https://www.statsmodels.org/)] and nonstandard packages [sliding1d, version 1.0 (https://github.com/gwappa/python-sliding1d); fitting2d, version 1.0.0a2 (https://doi.org/10.5281/zenodo.3782790)]. The Kruskal–Wallis test, followed by *post hoc* pairwise tests using Dunn’s test with Bonferroni’s correction, was performed using a custom Python script based on the one found at https://gist.github.com/alimuldal/fbb19b73fa25423f02e8.

#### Tracking whisker movement

Whisker positions were estimated using the custom Python script (videobatch; https://doi.org/10.5281/zenodo.3407666). The top-view videos first underwent a maximum intensity projection using the videobatch script. Regions of interest (ROIs) for tracking were selected manually for the C2 whiskers on both sides of the face using the Fiji freehand selection tool (Dominiak et al., 2019; Sehara et al., 2019). Using the Python script, pixels that belonged to a particular hue value were collected and the luma (brightness)-weighted average position was computed. For frames where the algorithm failed for any reason, values were dropped and were filled in later by linear interpolation.

The whisker positions tracked during each behavioral session were then converted to whisking angles. Using the fitting2d Python library, a circle was fit to the set of two-dimensional positions for each whisker, and the position at each time point was converted into the polar coordinates around the fitted circle.

The analysis of set point and amplitude of whisker motion in the Airtrack has been described in previous work by Dominiak et al. (2019). Here, taking into account this previously published work—and taking into account that (1) the motion of a whisker is determined by the motion of the whisker pad and the intrinsic muscles associated with individual whiskers; and (2) the set point and amplitude of whisker change in the course of single behavioral session in complex behavior state-dependent manner (Dominiak et al., 2019)—we used the angle corresponding to the session median position, defined as the provisional zero angle for each session. Deviations in whisker positions were represented as the percentage of the full range of deviation during each trial (i.e., between the trial minimum and the trial maximum angles).

Whisker asymmetry values were defined as the difference of normalized positions of the left whisker (*w*_Left_) and the right whisker (*w*_Right_). The value *w*_Right_ – *w*_Left_ was computed for a left-turning trial, whereas the value *w*_Left_ – *w*_Right_ was used for right-turning trials.

#### Tracking eye movements

DeepLabCut (version 2.1; Mathis et al., 2018; Nath et al., 2019) was used to track pupil positions from video frames. A deep-neural network model was trained to detect eight points on the edge of the pupil on each video frame. A single model was used for tracking pupils of both sides of the face of all animals in all sessions. In total, 284 frames from 28 sessions from different animals were manually annotated to train the network. For each video frame, the eight edge points of the eye detected by the deep-neural network were then fitted by an ellipse, using the fitting2d Python library. The position and the diameter of the fitted ellipse were considered to be those of the pupil.

Because the size of the eyes in the field of view of the video varied across behavioral sessions, the position of the pupil was normalized with respect to the size of the eye. For each eye during each behavioral session, the average eye shape was first delineated manually using the ROI Manager of ImageJ, based on the average-projection image of eye videos during the session. A parabola curve was then fitted to the trace corresponding to either the top or the bottom eyelid, using the fitting2d Python library. The two corners of the eye were then computed as the crossing points between the two fitted parabola curves. These defined the line segment representing the width of the eye. The position of the pupils was first projected onto the eye-width segment, and then was represented in terms of the fraction relative to the full width of the eye (hereafter called the normalized pupil position).

During analysis, pupil deviation values were defined as the average deviation of the normalized position of left and the right pupils in the direction of turn.

#### Detection of saccades

The first derivative (dX/dt) of normalized eye positions was computed to obtain the pupil speed. Sudden changes in eye position appeared as distinct spikes in the corresponding eye speed trace. Saccades were said to have occurred when the absolute values of eye speed for both eyes crossed a threshold value that was set empirically at 0.1% (relative to the full eye width) per frame. If a train of these high-speed events occurred within an interval of <250 ms, it was considered to be a processing artifact and was discarded from the analysis.

#### Normalization of behavioral state durations

The duration for each behavioral state was normalized using interpolation. For each behavioral epoch, we first set up a normalized time base such that the time points 0 and 1 marked the start and the end of the epoch. The data points were resampled from the original time base (i.e., frames) to the normalized time base using interpolation.

#### Analysis using receiver operating characteristic curves

We used receiver operating characteristic (ROC) curves to examine whether eye movement, whisker asymmetry, or both could predict the turn direction (Green and Swets, 1966). We divided the backward-movement behavioral epoch into five distinct time bins. For each time bin, we set up a set of classifier models. As its input, each classifier model received either a whisker asymmetry value (i.e., the right whisker protraction subtracted by the left whisker protraction) or an eye position value (positive values indicating leftward deviations and negative values indicating rightward deviation). Each model had a certain prespecified threshold value. When input value was above the threshold value, the model determined that a left turn would occur. If the input value was below the threshold value, the model predicted a right turn. We supplied the set of models of different threshold values with behavioral data (either whisker asymmetry or eye positions). For each time bin, the probability of correct prediction for each model of the upcoming turn direction to the left, the “true positives,” and the probability of predicting the upcoming turn direction incorrectly, the “false positives” (the model predicted to turn left, whereas the animal turned right in reality), were computed. The ROC curve for each time bin was then plotted based on these probabilities, using the set of models having different threshold values. The area-under-the-curve values generated by this analysis were compared with the shuffled data, and significance was assessed for each time bin, for whisker asymmetry and eye movement separately. Statistical comparison between the raw and shuffled data and between whisker-based and eye-based data in each time bin was performed using the Mann–Whitney *U* test. The Mann–Whitney *U* test with Bonferroni’s correction was used for pairwise comparison between data belonging to different time bins.

#### Analysis using perceptrons

We used single-layer perceptrons to examine the contribution of eye movement and whisker asymmetry in predicting the turn direction (Minsky and Papert, 1969). For this analysis, we divided the backward-movement behavioral epoch into five distinct time bins and set up classifier models. Here, for each time bin, we laid out individual time points in a two-dimensional space of whisker asymmetry and eye positions, and a line was fitted to separate left-turning and right-turning points as much as possible. Thus, the classifier predicted that the left turn or right turn would occur based on which side of the line individual points were located. The fidelity of separation was defined as the fidelity of prediction, as follows: fidelity marked 1.0 if the line correctly separated the points into left-turning and right-turning ones, and 0.5 if each side of the line contained equal numbers of left-turning and right-turning points.

For each time bin, we first prepared two models; the full model was prepared based on the true data points, whereas in the null model, both whisker asymmetry and eye positions were shuffled in relation to the turning directions. To compute the unique contribution of either whisker asymmetry or eye positions to the separation fidelity of the full model, we prepared a partial model based on the dataset where only a single selected variable was shuffled, and computed the resulting decrease in the separation fidelity compared with the full model. Statistical comparisons were performed using the Kruskal–Wallis test with Bonferroni’s correction by taking into account all of the four models of all the time bins.

### Data availability

All the raw videos and behavioral state annotation data used in this study are deposited at GIN (https://dx.doi.org/10.12751/g-node.j9wxqe).

## Results

### Whisking and eye position

We trained head-fixed mice to navigate on Airtrack, a real-world floating plus maze (Nashaat et al., 2016; Dominiak et al., 2019). In this task, mice started at the end of a lane, then backed out of the lane, and turned in the expected direction as indicated by an LED. If mice turned in the correct direction, entered a lane, and walked to the end of the lane, a lick spout descended for mice to lick. Mice have directional biases; they prefer turning to the left as they move backward and exit a lane. In the earlier work using these mazes, individual mice made left or right turns on every trial, almost exclusively (Dominiak et al., 2019).

To overcome this bias, as they navigate on Airtrack, we trained mice to attend to a go cue that indicated the expected turn direction for the trial (Fig. 1*A*,*B*). In the course of a session, mice had to turn left on some randomly selected trials and right on the other trials. Consistent with earlier work on these platforms, mice whisked when they were engaged in active behaviors, and they did not whisk much when they were inactive (Fig. 1*C*,*D*, Movie 1; Dominiak et al., 2019). In addition, whisking was asymmetric. In every trial, for a large fraction of each trial, as mice moved through the maze, they moved their right and left whiskers in opposite directions, retracting whiskers on one side and protracting them on the other side (Fig. 1*C*, top, left turn trial, bottom, right turn trial). The asymmetry of the whiskers reflected the direction of turn (Fig. 1*C*, top, left turning trial, bottom, right turning trial), with the right and left side C2 whiskers mirroring each other for the right and left turns. These observations suggested that mice position their whiskers asymmetrically as a part of their motor plan.

In addition to positioning the whiskers asymmetrically and whisking, mice also moved their eyes on every trial (Fig. 1*C*, Movie 1). They performed saccade-like, abrupt and intermittent fast eye movements when they were moving (i.e., when they were engaged in active behavior). The movement/position traces of both eyes were similar (Fig. 1*C*, red and blue traces); mice moved their eyes conjugately, bilaterally, to the same extent, at approximately the same time, in the direction of the upcoming turn. Consequently, right and left turn trials elicited movement of both eyes, but the movement was in the opposite direction for the two turn directions. Once mice stopped moving—when they were expecting a reward, licking, or were waiting to begin the new trial—movement of the eyes diminished greatly.

**Figure 2.**
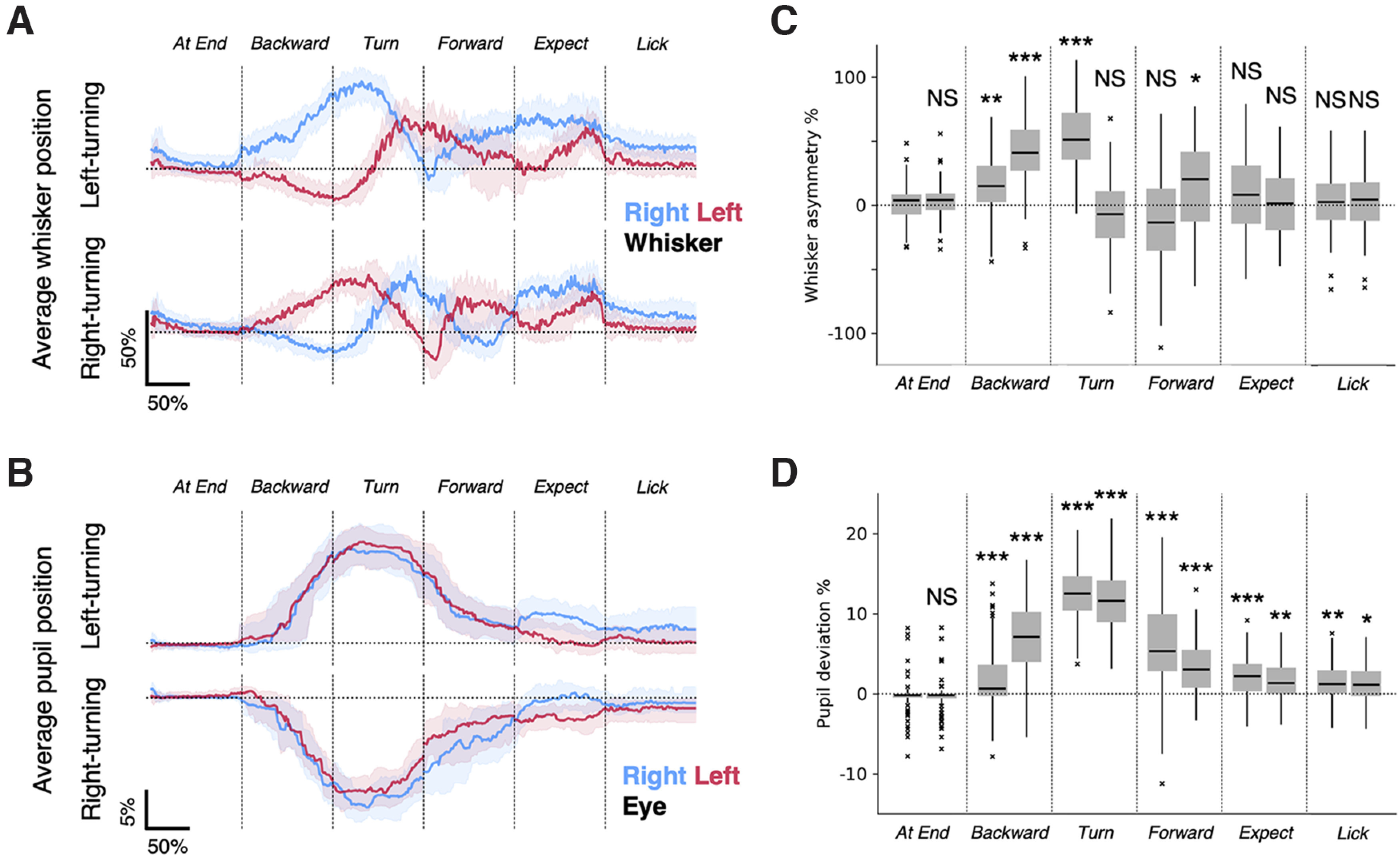
Whisker asymmetry and eye position in relation to the cued direction of movement. ***A***, Averages of whisker positions. Median positions for the left whisker (thick red line) and right whisker (thick blue line) show that whisker asymmetry was related to the direction of movement, and was evident early in the backward movement of mice out of the lane. The shaded areas show the 25th to 75th percentiles. The asymmetry between whiskers emerges at the beginning of backward movement, and reverses once during the course of the turn and a second time during the forward motion. Asymmetry vanishes when mice wait for and expect reward. Note that whisker asymmetry on right-turn and left-turn trials are almost mirror images of each other. ***B***, Averages of eye positions. Median positions for the left eye (thick red line) and the right eye (thick blue line) move conjugately in the behavioral state-specific manner. The shaded areas show the 25th to 75th percentiles. Note that the average eye positions on right-turn and left-turn trials are inverted images of each other. Whisker and eye positions were normalized for amplitude (see Materials and Methods) and for time (because trial durations varied; see Materials and Methods). The numbers of behavioral epochs used were as follows: At End, *n* = 54 (left-turning) epochs and *n* = 36 (right-turning) epochs; Backward, *n* = 54 (left-turning) epochs and *n* = 36 (right-turning) epochs; Turn, *n* = 52 (left-turning) epochs and *n* = 40 (right-turning) epochs; Forward, *n* = 63 (left-turning) epochs and *n* = 50 (right-turning) epochs; Expect, *n* = 51 (left-turning) epochs and *n* = 38 (right-turning) epochs; Lick, *n* = 51 (left-turning) epochs and *n* = 36 (right-turning) epochs. ***C***, ***D***, Tukey box plots of average whisker asymmetry (***C***) and eye deviation (***D***) during different behavioral epochs. *p* < 0.0001, Kruskal–Wallis test. *Post hoc* pairwise tests were performed to compare with the first half of the end-of-lane epoch, using Dunn’s test with Bonferroni’s correction. ***p < 0.001, **p < 0.01, *p < 0.05, NS, *p* > 0.05.

To quantify the effects of the behavioral state of the animal on whisker and eye positions, we normalized the data for time (each trial and each behavioral epoch could have a different duration) and normalized whisker position and eye position data for the extent/amplitude of movement (Fig. 2). These normalized average traces confirm what the single-trial data show: the side-to-side whisker asymmetry (Fig. 2*A*) and eye movement (Fig. 2*B*) were related to the behavioral state of the animal and the direction of turn in the maze. When right-turn trials were compared with left-turn trials, whisker asymmetry appeared as a mirror image, and eye movement traces appeared as inverted mirror images of each other. Whiskers on two sides of the face moved bilaterally but in the opposite directions, and at the same time mice moved their eyes conjugately in the direction of the upcoming turn. This pattern of whisking and eye movement were evident when mice were in an active state (i.e., when the animals were moving backward, forward, or turning), and the pattern was not evident when mice were standing still at the end of a lane, expecting reward or licking. When mice were active and moving backward or turning, in both right and left turning trials, there was significant whisker asymmetry and significant change in eye position compared with behavioral epochs where mice were just standing still (Fig. 2*C*,*D*). Together, these data indicate that whisker asymmetry and the movement of the eyes were active processes occurring in a behaviorally relevant manner as mice navigated the plus maze.

### Predicting turn direction with whisking and eye movements

**Figure 3.**
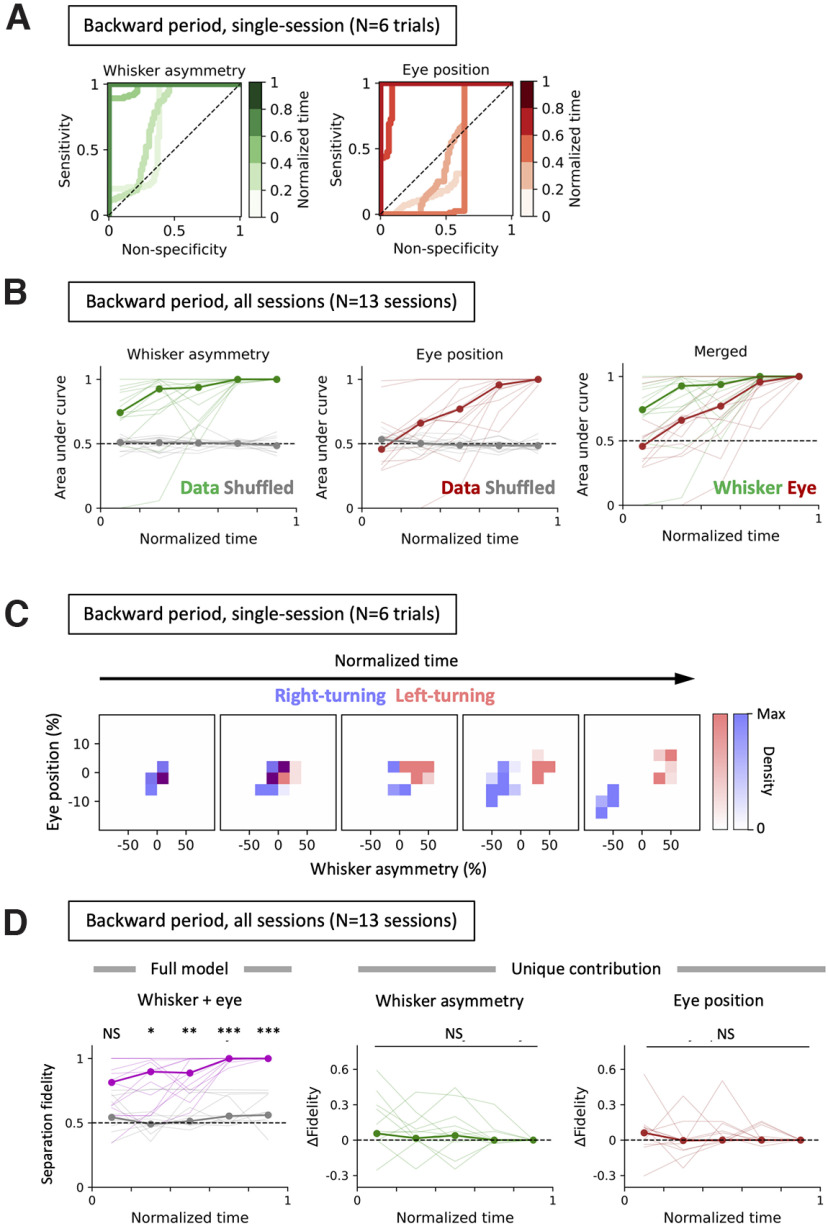
Behavior during the backward motion predicts the next turn direction of the animal. ***A***, ROC curves were generated based on the whisker asymmetry (left, green) and the eye position (right, red) during the period when the mouse was running backward along the lane to predict whether the animal was going to turn in the left or right. The denser curves represent the later phases in the backward movement epoch. Note that during the later phases, the curves are positioned closer to the top-left corner of the bounding square, indicating that whisker asymmetry and the eye position are good predictors of the upcoming turn direction. Data from a representative session are shown. ***B***, The AUC at different phases during the backward movement epoch in multiple behavioral sessions and animals (*n* = 17 sessions taken from 4 animals, consisting of 90 backward movement epochs in total) show that the whisker positions and eye positions work separately as an efficient predictor of the upcoming turn direction of the animal. The median AUC values were computed for whisker asymmetry (left, thick green line) or for the eye position (center, thick brown line). The gray lines on the two left panels represent the AUC values when the whisker asymmetry or the eye position data were randomized within the backward period. The right panel shows the comparison of predictive performances between the whisker asymmetry and the eye position on different phases within the backward movement. Pairwise comparisons indicate that whisker asymmetry-based prediction (left) was significantly better for later time bins than for earlier ones, reaching significance midway through backward motion, as follows: bin #1 versus bin #2, *p* = 1.0000; bin #1 versus bin #3, *p* = 0.9037; bin #1 versus bin #4, ***p = 0.0007; bin #1 versus bin #5, ***p = 0.0002; bin #2 versus bin #3, *p* = 1.0000; bin #2 versus bin #4, *p = 0.0387; bin #2 versus bin #5, **p = 0.0016; bin #3 versus bin #4, *p = 0.0137; bin #3 versus bin #5, ***p = 0.0005; bin #4 versus bin #5, *p* = 0.3735; Mann–Whitney *U* test with Bonferroni’s correction. Pairwise comparisons for eye movement (center) also indicate that late in the backward motion eye movement was a good predictor of upcoming turn direction, as follows: bin #1 versus bin #2, *p* = 1.0000; bin #1 versus bin #3, *p* = 0.2395; bin #1 versus bin #4, **p = 0.0032; bin #1 versus bin #5, ***p = 0.0001; bin #2 versus bin #3, *p* = 1.0000; bin #2 versus bin #4, *p = 0.0316; bin #2 versus bin #5, ***p = 0.0009; bin #3 versus bin #4, *p* = 0.4514; bin #3 versus bin #5, **p = 0.0033; bin #4 versus bin #5, *p = 0.0453; Mann–Whitney *U* test with Bonferroni’s correction. Pairwise comparisons between whisker asymmetry versus eye positioning (right) indicate that whisker asymmetry is a better predictor of the upcoming turn direction in the early phases of backward movement period: bin #1, *p = 0.0293; bin #2, *p = 0.0269; bin #3, *p* = 0.2799; bin #4, *p = 0.0275; bin #5, *p* = 0.3560; Mann–Whitney *U* test. ***C***, Two-dimensional distribution of whisker position and eye movement before left (red) and right (blue) turns. The histograms of whisker asymmetry and eye movement are plotted relative to each other over five temporal bins as mice move backward in a lane. The heights (densities) of the histograms were scaled for visualization, and time was normalized over the time periods during backward movement. Over time as the mice move backward and closer to the turn, there was a marked separation between data points from right-turning and left-turning trials. ***D***, Fidelity of separation. The status of the separation was examined by drawing a line in the two-dimensional space that best separated the two sets of data points. The fidelity of separation (i.e., of predicting the left turning directions based on separation using that line) was computed. Left, The magenta line indicates the fidelity when true whisker and eye data were provided, and the gray line represents the results from the null models, where both whisker and eye data were shuffled relative to the direction of turn. Later bins during the backward period have significant fidelity in separation (bin #1, *p* = 0.3674, NS; bin #2, *p = 0.0379; bin #3, **p = 0.0097; bin #4, ***p = 0.0000; bin #5, ***p = 0.0000; Kruskal–Wallis test with Bonferroni’s correction). Center and right: unique contribution of whisker asymmetry (center) and eye positions (right) to the fidelity of separation. Shuffling the whisker asymmetry or eye positions alone has no significant additional effect on the fidelity of the separation compared with the full model (NS, *p* = 1.0 for all time bins, Kruskal–Wallis test with Bonferroni’s correction).

We used ROC curves to examine whether eye movement, whisker asymmetry, or both could predict the turn direction (Fig. 3; see also Materials and Methods). When normalized for time (during the backward movement epoch) and binned, the ROC curves reveal that both the whisker asymmetry and eye position become increasingly stereotyped and increasingly predict the turn direction (Fig. 3*A*).

While both eye movement and whisker movement accurately predicted turn direction before the animal started to turn the maze, examination of the area under the curve (AUC) across behavioral sessions (*n* = 13 sessions taken from four animals, consisting of 77 backward movement epochs in total) showed that the AUC for models based on whisker asymmetry was significantly larger than the chance level from the very beginning of the backward-movement period (bin #1, **p = 0.0066; bin #2, **p = 0.0029; bin #3, ***p = 0.0000; bin #4, ***p = 0.0000; bin #5, ***p = 0.0000; Mann–Whitney *U* test; Fig. 3*B*, left). This implied that whisker asymmetry was a good predictor of the upcoming turn direction. Pairwise comparisons indicate that whisker asymmetry-based prediction was significantly better for later time bins than for earlier ones, reaching significance (*p* < 0.01, Mann–Whitney *U* test with Bonferroni’s correction) midway through the backward motion (Fig. 3*B*, left).

Except for the beginning of the backward movement, eye position also predicted the upcoming turn direction (bin #1, *p* = 0.6444; bin #2, *p* = 0.1118; bin #3, **p = 0.0029; bin #4, ***p = 0.0000; bin #5, *p* = 0.0000; Mann–Whitney *U* test). As the animal moved closer to the turn, during or just after the mid-point of the backward motion, eye position became a good predictor of the upcoming turn direction (*p* < 0.05, Mann Whitney *U* test with Bonferroni’s correction; Fig. 3*B*, middle).

Additionally, models based on whisker asymmetry were slightly but significantly better at predicting turn direction — just as the animal started the backward movement — than those using eye positions (bin #1, *p* = 0.0293*; bin #2, *p* = 0.0269*; bin #3, *p* = 0.2799; bin #4, *p* = 0.0275*; bin #5, *p* = 0.3560; Mann–Whitney *U* test; Fig. 3*B*, right).

We then asked whether having both whisker asymmetry and eye movement together contributes to a higher predictive power than having single behavioral variables alone. When histograms of whisker asymmetry and eye movement were plotted relative to each other over the five temporal bins as mice move backward in a lane, there was a marked separation between data points from right-turning and left-turning trials (Fig. 3*C*). The fidelity of separation for predicting the turn directions was examined for whisker and eye position. In later bins, the model using both eye and whisker movement data was significantly better at prediction, than the model based on the data where both of the input parameters were shuffled in relation to turning directions (bin #1, *p* = 0.3674; bin #2, *p = 0.0379; bin #3, **p = 0.0097; bin #4, ***p = 0.0000; bin #5, ***p = 0.0000; Kruskal–Wallis test with Bonferroni’s correction; Fig. 3*D*, left). Shuffling the whisker asymmetry (Fig. 3*D*, middle) or eye positions (Fig. 3*D*, right) had no significant additional effect on the fidelity of the separation compared with the full model (*p* = 1.0 for all time bins, Kruskal–Wallis test with Bonferroni’s correction).

Together, these analyses reveal that the information in whisker asymmetry and eye position, individually and together, were sufficient to predict the turn direction before the animal begins to turn. The results of our analysis also imply that whisker asymmetry and eye movement contain largely overlapping information in predicting the turn direction.

### Saccadic eye movement and behavioral state

**Figure 4.**
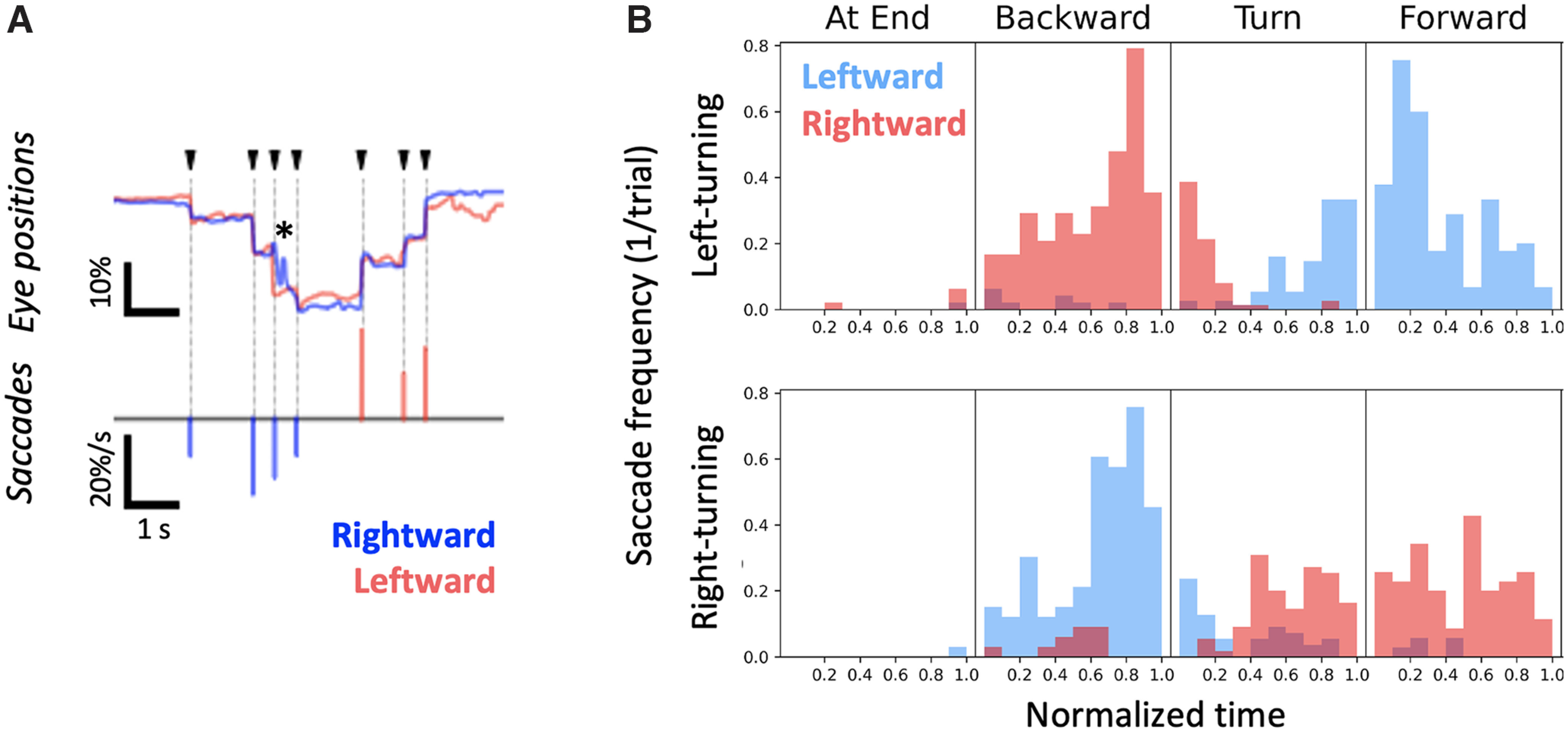
Saccadic eye movements related to behavioral state and turn direction. ***A***, Detection of saccades. Saccadic events were defined as the time points when the two eyes (top, red and blue traces) moved rapidly and conjugately (arrowheads). Asterisk refers to an occlusion-related artifact when one eye was partially occluded by the movement of a wall. The bottom plot shows the detected saccadic events. The events were considered rightward (blue bars) or leftward (red bars) based on their directions of movement. The height of each bar indicates the size of the corresponding saccadic event. ***B***, Timings of saccades were related to the behavioral state, and saccade direction was related to turn direction. Histograms of per-epoch saccade occurrences were generated based on the data of 91 trials in 17 sessions taken from 4 animals. Leftward saccades (red bins) occurred as the mice turned leftward (top distribution), when they backed out of a lane; and rightward saccades (blue bins) occurred when they turned right and went forward into a lane. The direction of saccades was inverted for right-turning trials (bottom distribution). Saccades were detected mainly when animals were moving and active. The timings of saccades were distributed broadly across different phases during each behavioral epoch.

While the eye movement data from single trials shows that head-fixed mice moved their eyes in a stepwise fashion on each trial (Fig. 1), this fast movement of the eyes tended to be smoothed out in the average traces (Fig. 2). To examine the relationship between the rapid saccadic eye movements and behavioral state, we related behavioral states to saccades, which appeared as distinct spikes in the corresponding eye speed trace (Fig. 4*A*). Eye movements were counted as saccades if the following criteria were met: absolute value of eye speed for both eyes was above the threshold of an absolute eye speed of 0.1% of full eye-width per frame; and there was no additional spike of higher absolute speed related to eye movement within 250 ms of the saccade. The duration of each behavioral epoch (end of lane, backward movement, left or right turns, and forward movement) was normalized and divided into 10 equal bins, and the number of saccades that occurred within each time bin was counted. This analysis revealed that mice made saccades predominantly in the latter half of the backward movement, as they moved out of the lane. Saccades also occurred as mice turned and went forward into an adjacent lane (Fig. 4*B*). Consistent with the analysis of eye positions (Figs. 1*C*, 2), saccades were more likely to occur in the direction of the upcoming turn, rather than in the opposite direction. The distribution of saccades suggests that mice look in the direction of turn as they exit backward out of the lane, and again as they enter another lane. Note that, although saccades occurred primarily when the mice were close to a turn or turning, saccades also occurred when the animals were merely moving forward or backward in a lane.

### Correlation between saccades and whisker asymmetry

**Figure 5.**
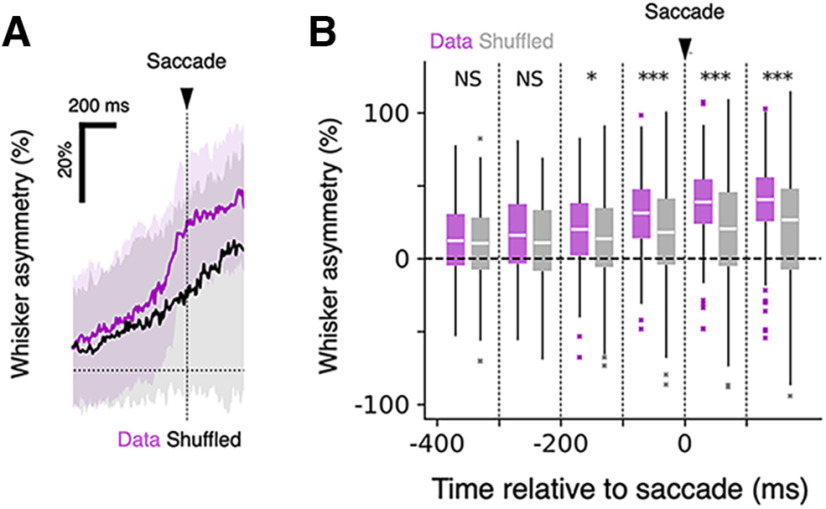
Coordination between saccadic movement and whisker asymmetry. ***A***, Changes in whisker asymmetry in relation to the occurrence of saccades around the onset of movement during the backward movement period. The dashed line at zero, and the black triangle, mark saccade onset. On average, whisker asymmetry increased 100–200 ms before the time the eyes moved (*n* = 120 leftward saccade events and *n* = 94 rightward saccade events). The increased asymmetry was maintained for hundreds of milliseconds after each saccade. The correlograms based on the original data (magenta) and the data where the timings of saccade events were shuffled within the backward movement period in the trial (black) are shown. Solid lines indicate the median traces, and the shaded regions indicate the intervals between the 25th and 75th percentiles. ***B***, Quantification of changes in whisker asymmetry. The median whisker asymmetry values of 100 ms bins were computed for each trace. The arrowhead indicates the saccade onset. Tukey box plots were generated based on the original data (magenta) and the shuffled data (gray). The distribution of values were compared in the bin-to-bin basis between the original and the shuffled datasets. ***p < 0.001, *p < 0.05, NS, *p* > 0.05; Mann–Whitney *U* test, *n* = 214 saccade events.

In many species, eye movement is correlated with head movement (Land, 2006). In rodents, head movement has often been related to both eye and whisker movement (Hartmann et al., 2003; Towal and Hartmann, 2006; Meyer et al., 2018). But mice can move their whiskers and eyes independently of their heads, even when they are head fixed, so it was possible that, although movement of the head was prevented, eye and whisker movements could be coordinated. To examine the relationship between saccades and whisker movement, we plotted the level of asymmetry before and after saccadic eye movements as mice moved backward out of the lane (Fig. 5*A*). Our analysis revealed that as mice moved backward and moved their eyes, they also changed how they positioned their whiskers. Whisker asymmetry increased measurably and significantly, 100–200 ms before mice made saccades during their backward movement (Fig. 5*B*). These results suggest that mice coordinate eye movement with asymmetric positioning of the whiskers as part of the motor plan and execution of the movement related to the turn.

## Discussion

**Figure 6.**
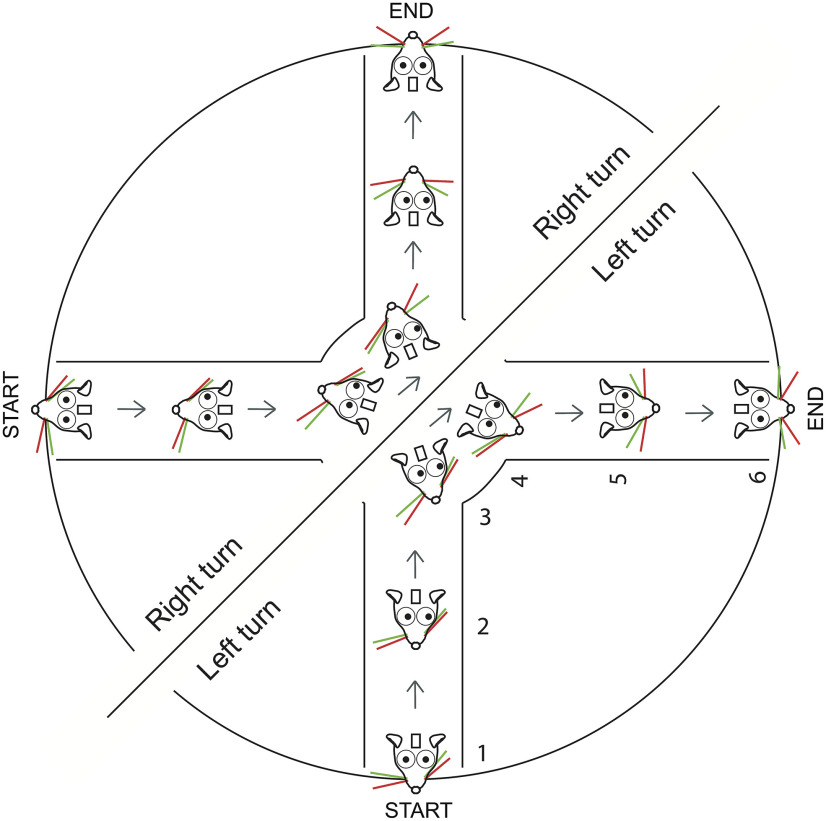
Schematic of eye movement and whisker asymmetry in the course of right and left turns. Whisker asymmetry and eye movement in the course of single right or left turns.

To behave means to plan and emit a sequence of actions (Tinbergen, 1955; Krakauer et al., 2017). On one hand, the behavior we used here was complex; mice were head fixed as they moved their entire body through a “real-world” maze, as they pushed and rotated the platform around themselves, and entered or exited lanes. On the other hand, the behavior was simple; mice were in an environment that they controlled. The environment had lanes, similar to the burrows that mice tend to live in, in their natural habitat. All that mice had to do was to keep track of a cue that indicated the direction to move in, to recognize an adjacent rewarding lane, and to enter it. There was no time pressure, no requirement for mice to discriminate one thing from another. The goal was just to forage for water by moving in the cued direction. In the course of this behavior, as part of their motor plan, mice moved their whiskers and eyes in anticipation, in preparation for a turn in the maze. The bilateral positioning of the whiskers and eyes predicted the turn direction mice expected to impose on the maze (Figs. 3, 6).

In this maze, mice moved their body, and, while they moved their body, they also moved their eyes and whiskers bilaterally. In fact, in the course of this behavior, mice even move their nose and reset the set point of their whiskers, presumably by moving their whisker pad (Dominiak et al., 2019). One key difference between the earlier work and this work is that here mice had to attend to cues, and move in one of two directions based on a cue. In the earlier work, mice mostly moved in their preferred direction, right or left, but not in both directions. Here we trained mice to overcome their bias by using directional cues, and we found that from trial to trial, the bilateral whisker movement reflected turn direction instead of an innate preference. We also found that the retraction and protraction of whiskers were coordinated with the direction of eye movement.

Eyes and whiskers move similarly in a look-ahead function. Eye movement could be used for assessing the distance to a wall or to an opening. Whiskers could be positioned to anticipate the upcoming turn. But whether mice innately coordinate these movements, or they first learn to coordinate the movement of eyes and whiskers/face when they learn to navigate the maze, is not clear. Whether mice coordinate whisker and eye motion in their natural, freely behaving condition is also not known, in part because the entire behavioral repertoire that we have measured here is not easily or reliably measured in freely moving animals. We also do not know whether circuits for eye movement, whisker movement, motor planning, and body movement are all active simultaneously in adjacent parts of anterior frontal and sensorimotor cortices, and how activity in these circuits interacts with subcortical circuits to generate the sequence of movements.

### Eye movement in mice

Rodents use vision to locate objects and to avoid airborne (Morris, 1979) or ground-dwelling predators (Doncanster et al., 1990). Freely behaving mice move their eyes when they move their head; they move their head and eyes to monitor looming stimuli and to coordinate freezing or escaping behaviors (De Franceschi et al., 2016). One unique feature of freely moving rodents is that they move their eyes in both conjugate and disconjugate fashions (Sakatani and Isa, 2007; Wallace et al., 2013; Wang et al., 2015; Payne and Raymond, 2017; Meyer et al., 2018; Samonds et al., 2018). When rodents are head fixed, the frequency of eye movement is reduced and eye movement becomes almost completely conjugate (Wallace et al., 2013; Samonds et al., 2018).

Our work here confirms the earlier work, showing that head-fixed mice do indeed move their eyes, and they move their eyes conjugately. We extend the earlier work in two ways; first, we show that although mice are not instructed to move their eyes (Itokazu et al., 2018; Sato et al., 2019), mice move their eyes reliably, in a behaviorally relevant fashion just before and during turns. Mice moved their eyes in anticipation of the turn, in the direction of the turn on every trial. Second, we show that eye movement was embedded in the coordinated motion of the animal. When combined with earlier work, this work shows that when mice move, they move their eyes and whiskers in a coordinated fashion, in the same direction. In this floating real-world maze, movement of the eyes was part and parcel of the concerted movement of the face and body. Currently, we do not know whether mice move their eyes and whiskers in a similar fashion in virtual reality systems that have mazes, or virtual reality systems that have visual streaming built into them, but this should be an avenue of future investigations.

### Sensory–motor coordination in the real-world environment

Simultaneous and continuous coordination of movement and sensation is part of the natural function of the brain (Welker, 1964; Wolpert and Ghahramani, 2000; Llinas, 2001; Wolpert and Landy, 2012; Musall et al., 2019; Stringer et al., 2019; Kaplan and Zimmer, 2020; Tantirigama et al., 2020). In their natural state, when animals move, they interact with their environment in multiple sensory–motor modalities; their limbs touch the floor, they look in the direction of the motion, their breathing changes, and, in the case of rodents, they move their whiskers. The movement of the animal changes the sensory scene for the animal, which in turn generates a new set of sensory stimuli (i.e., pressure on limbs, the novel texture under their skin, an updated stream of visual input, and tactile input from the whiskers) guiding mice around obstacles around and in front of them. These changing sets of stimuli elicit activity in the brain and are then used to reinforce the ongoing behavior or to modify the movement of the animal (Arkley et al., 2014; Kurnikova et al., 2017). As animals learn to navigate an environment, they can plan ahead, and anticipate what to expect before the actual sensory input occurs (Keller and Mrsic-Flogel, 2018).

It should be noted that head fixation is a key limitation of our work. When rodents are free to move, their eye movements are richer and more complex than in the head-fixed state (Wallace et al., 2013; Meyer et al., 2020). Consequently, the interaction between whisking and eye movements is expected to be much richer in the freely moving mouse. But it has not been possible to study this interaction in freely moving animals, so how the movement of each eye relates to the movement of whiskers on that side of the face in the freely moving animal, when eyes move disconjugately, is not known. Additionally, nothing is known about the interaction between the visual sensory–motor and vibrissal sensory motor systems as mice navigate their environment.

Another limitation of our work is the relatively small sample size. While this may have affected the statistical power in examining how much information is shared between whisker positioning and eye movement in predicting the planned motor behavior, it should be noted that, in the course of navigating the plus maze, mice position their whiskers asymmetrically and move their eyes in the direction of the upcoming turn on every trial. Whether the two behavioral variables encode information independent of each other would be the subject of future studies.

Our work sheds light on the innate strategy used by mice, and shows how they spontaneously, almost automatically, plan and coordinate the movement of their eyes, whiskers, and body. The floating plus maze we used here is likely to engage widespread cortical and subcortical circuits in a manner that is close to what the brain has evolved to do; that is, to move the animal through the world.

## Synthesis

Reviewing Editor: David Schoppik, New York University - Langone Medical Center Decisions are customarily a result of the Reviewing Editor and the peer reviewers coming together and discussing their recommendations until a consensus is reached. When revisions are invited, a fact-based synthesis statement explaining their decision and outlining what is needed to prepare a revision will be listed below. The following reviewer(s) agreed to reveal their identity: Jean Laurens, Ugne Simanaviciute.

Major remarks:

Throughout the study, the authors should describe the visual environment much more thoroughly since it is expected to have a strong influence on eye movements. In particular:

- What is the colour of the walls of the maze? The manuscript doesn’t describe them but in Video 1 they appear to be transparent. This would explain why the authors don’t observe dis-conjugate eye movements that would track the walls. This should be very clear in the Methods and in Figure 1.

- What is the height of the wall relative to the eyes? This will determine how much of the walls are in the visual field.

- Is there an ambient illumination in the room? Is there anything visible above the mouse? If the mice can see anything other than the wall, then these visual features will be fixed relative to the head and will tend to suppress eye movements.

- Where exactly are the LEDs (that indicate the direction of the turn) placed relative to the head? If they are placed laterally and the mice attend to them, it may influence their eye movements too.

-Figure 2 and Figure 4: In Figure 4, the authors point out that averaging eye position data (in Figure 2) smoothes out fast eye movements. This is correct! Therefore, the diagram in Figure 2B gives a wrong impression that eyes move smoothly. The authors should probably merge Figure 2 and Figure 4, so that this point is addressed right away.

Animal numbers: the Methods section states that you used 9 animals, but data analysis includes only 4 animals. What were the exclusion criteria?

-In the Abstract, Significance Statement, and Introduction, please highlight the novelty / contribution of this study

Minor remarks:

-Introduction Please talk about findings from Dominiak et al. in more detail, and explain what the exact gaps in knowledge are.

-Methods are a little complicated and might benefit from a figure showing the timeline of when what was done and how long it took, especially including the sample numbers (or dropout) from each stage.

-TTL abbreviation not explained.

-ROC curves abbreviation not explained, not referenced.

-There should be a justification for chosen recording speed for the movements recorded (fps). Most current research use much faster filming to properly image the whiskers (>500fps). Filming resolution should also be stated.

-Justify the number of whiskers tracked. One per side of the face is very low compared to other studies.

-A proper definition and description of whisker positions is missing. Where is the zero of the polar coordinates? Why have you chosen this, compared to the usual measure (i.e. angle of whisker with mouse’s cheek or midline of the head)? Please justify your choice in the text.

-Explain “luma-weighted”.

-How do you statistically control for repeated measures for the same animal?

-Results could be more consistent with the p value reporting and the spacing, some numbers are missing “p=”.

-Can you explain the difference of left and right-turning epochs, why is n of epochs so much larger in left turns? Were there any criteria for data selection? Also please address the number of trials and number of mice.

-References Dominiak et al. (2019) is missing from the reference list, check others too.

-Figures Figure 6 shows mostly the same as the video included and it has also been shown in Dominiak et al., 2019, so if you are short on space, could be removed.

-Introduction: mice also use their visual system to chase prey, see e.g. Hoy et al. Curr Biol 2016.

-Methods: the authors should briefly recapitulate the “plus maze” task. For a reader who is not familiar with the task, it is almost impossible to understand it without referring to previous publications.

-Methods: in the video, there seem to be some tactile cues attached to the wall of the maze. Please indicate it in methods and represent them on Figure 1.

-Results, p12, second paragraph, second sentence: “They moved their eyes rapidly”. In here, the authors refer to saccadic eye movement, but in general “rapidly moving the eyes” can mean a lot of different things (VOR, OKN, etc). Please be specific.

-Results, p14: it would be interesting to know if eye movements predict turn direction independently of whisker movements. Have you considered using a model that includes both variables, and/or performing a partial correlation analysis?

-Discussion, first paragraph: “to recognize an adjacent dark lane”. What do you mean?

-Discussion is missing a limitations section to discuss the limitations of your work, including - head fixing and sample sizes.

## Author Response

To the editor,

We have revised the manuscript in accordance with the reviewer’s comments. Please see our detailed response below.

Sincerely,

The authors

---------------------------------------------

Synthesis of Reviews:

Significance Statement Comments for Author (Required):

Please highlight the contribution of this work more clearly

Comments on the Visual Abstract for Author (Required):

N/A

Synthesis Statement for Author (Required):

Major remarks:

Throughout the study, the authors should describe the visual environment much more thoroughly since it is expected to have a strong influence on eye movements. In particular:

We have added details about the visual environment in the Methods section (lines 89-99).

- What is the colour of the walls of the maze? The manuscript doesn’t describe them but in Video 1 they appear to be transparent. This would explain why the authors don’t observe dis-conjugate eye movements that would track the walls. This should be very clear in the Methods and in Figure 1.

We used black opaque or red plexiglass walls. The difference in materials had no effect on any aspects of the behavior. We have added that point to the Materials and Methods section (lines 89-91). Please note that the colors in Video 1 appear differently from what they were in reality because of the aberrant white-balance settings used with the UV illumination.

- What is the height of the wall relative to the eyes? This will determine how much of the walls are in the visual field.

The walls were 2.5 cm, and the top of the mouse head was under the walls. We have added the description in the Materials and Methods section (lines 89-91, lines 96-97).

- Is there an ambient illumination in the room? Is there anything visible above the mouse? If the mice can see anything other than the wall, then these visual features will be fixed relative to the head and will tend to suppress eye movements.

The UV (365-400 nm) light (used to illuminate and track whiskers) provided some ambient illumination. The mouse could move its eyes to see the apparatus around it, but the entire apparatus was covered with a black drape to prevent mice from seeing any additional objects in the environment. Mice could see the visual cue, with the LED positioned in front of their eyes. We have added more detailed description in the Materials and Methods section (lines 93-99).

- Where exactly are the LEDs (that indicate the direction of the turn) placed relative to the head? If they are placed laterally and the mice attend to them, it may influence their eye movements too.

They are placed laterally at 3.5 cm from the lick-spout, in front of the animal’s eyes. We added the description in the Materials and Methods section (lines 93-94). As the reviewer notes, it is possible that the existence of the LEDs may have affected the animal’s eye movements. But our work shows that the eyes move at particular time in the trial, and in relation to upcoming turn direction. The positions of indicator LEDs are fixed relative to the animals’ eyes, so they cannot explain this observed eye movement.

-Figure 2 and Figure 4: In Figure 4, the authors point out that averaging eye position data (in Figure 2) smoothes out fast eye movements. This is correct! Therefore, the diagram in Figure 2B gives a wrong impression that eyes move smoothly. The authors should probably merge Figure 2 and Figure 4, so that this point is addressed right away.

While it might be better to merge the two figures, our point in Figure 2 was to compare the average movement of whiskers and eyes with respect to the animal’s behavioral states. This point is of importance to the current study, and we want to emphasize it separately from the existence and the analysis of saccadic eye movements. We revised our Results section to make our intentions clear (line 316). Note also that Figure 1 shows the saccades, the raw data of eye positions and here we have rewritten the text slightly to emphasize saccade-like movements. In addition, as the reviewers noted, Figure 4 includes analysis of the saccades.

Animal numbers: the Methods section states that you used 9 animals, but data analysis includes only 4 animals. What were the exclusion criteria?

We have removed any mention of 9 animals from the manuscript. Nine animals were used for the overall behavioral performance of mice, but data from most of these animals has not been used here. The main rationale for excluding animals and trials was the tediousness of manual annotation and the inability of some mice to overcome their turn direction bias / perference. We removed the mention of the unused trials in the Materials and Methods section (lines 130-136).

-In the Abstract, Significance Statement, and Introduction, please highlight the novelty / contribution of this study

We have revised the manuscript in accordance with the reviewer’s comment (lines 13-17, 20-27, 57-64).

Minor remarks:

-Introduction Please talk about findings from Dominiak et al. in more detail, and explain what the exact gaps in knowledge are.

We have fixed this in the introduction (lines 57-64).

-Methods are a little complicated and might benefit from a figure showing the timeline of when what was done and how long it took, especially including the sample numbers (or dropout) from each stage.

We have added more detail in the methods and added Table 1 to describe the sample numbers (lines 130-136, 760-761).

-TTL abbreviation not explained.

We have added the explanation (line 154).

-ROC curves abbreviation not explained, not referenced.

We have added the explanation and reference to Green and Swets,1966 (lines 237-239).

-There should be a justification for chosen recording speed for the movements recorded (fps). Most current research use much faster filming to properly image the whiskers (>500fps). Filming resolution should also be stated.

Our goal was to capture whisking movement which in mice is on the order 20-25 Hz. A frame rate of 200 Hz is well above Nyquist frequency and is sufficient for this. Furthermore, Dominiak et al., showed that the tactile events, the very high frequency slip stick events do not explain the behaviors that are at the core of this work. Mice perform the behavior even after whiskers have been trimmed off, and mice move their whiskers in a similar manner after whiskers have been trimmed off. Also note that eye movements occur at lower frequencies at least in head fixed mice. Filming resolutions were added in the Materials and Methods section (lines 138, 148).

-Justify the number of whiskers tracked. One per side of the face is very low compared to other studies.

This work follows on the work by Dominiak and colleagues, which showed that even though it was possible to track more than one whisker, a single whisker tracked on each side of the face was sufficient to determine both setpoint and amplitude of whisker movement. Please also note that many investigators just track a single whisker on one side of the face, or track all whiskers without specifying what happens to a specific whisker.

-A proper definition and description of whisker positions is missing. Where is the zero of the polar coordinates? Why have you chosen this, compared to the usual measure (i.e. angle of whisker with mouse’s cheek or midline of the head)? Please justify your choice in the text.

Our choices follow on the work Dominiak et al. We have added details to the Materials and Methods section (lines 190-199).

-Explain “luma-weighted”.

We have added a description (lines 183-184).

-How do you statistically control for repeated measures for the same animal?

In this task mice show the same whisker and eye movement pattern on every trial of the same kind (same turn direction). The main variable is the turn direction. There can be timing and duration differences from trial to trial or animal to animal, but on each trial mice move their whiskers asymmetrically and move their eyes in the direction of the upcoming turn. We make use of the reliability and robustness of this behavior.

-Results could be more consistent with the p value reporting and the spacing, some numbers are missing “p=”.

We have corrected this point.

-Can you explain the difference of left and right-turning epochs, why is n of epochs so much larger in left turns? Were there any criteria for data selection? Also please address the number of trials and number of mice.

Mice prefer moving to the left, they have a pronounced inherent preference. It is much harder to make mice turn to the right, even with conditioning, bias correction or visual cues. Because the mice could make as many turns as they like before turning in the right direction, one trial can contain both left and right turns. During the analysis, we picked up the time periods (no matter whether they are found at the beginning of a trial or not) when the animal was sitting still at the end of the lane, moving backward along the lane, and then exiting out of the lane to make a turn.

-References Dominiak et al. (2019) is missing from the reference list, check others too.

We have added Dominiak et al and checked all the items in the reference list.

-Figures Figure 6 shows mostly the same as the video included and it has also been shown in Dominiak et al., 2019, so if you are short on space, could be removed.

This Figure additionally summarizes eye movements and their relationship to whisker movements as mice navigate through the maze.

-Introduction: mice also use their visual system to chase prey, see e.g. Hoy et al. Curr Biol 2016.

We have cited the Hoy paper now.

-Methods: the authors should briefly recapitulate the “plus maze” task. For a reader who is not familiar with the task, it is almost impossible to understand it without referring to previous publications.

We have added more detail in the Results section (lines 284-287). In addition, we have separated the “plus-maze task” section from the training section in Materials and Methods to be clearer on this point (lines 104-113).

-Methods: in the video, there seem to be some tactile cues attached to the wall of the maze. Please indicate it in methods and represent them on Figure 1.

We have added more detail in the description of the methods (line 159) and legend (lines 678-679).

-Results, p12, second paragraph, second sentence: “They moved their eyes rapidly”. In here, the authors refer to saccadic eye movement, but in general “rapidly moving the eyes” can mean a lot of different things (VOR, OKN, etc). Please be specific.

We have rewritten this (lines 381-382).

-Results, p14: it would be interesting to know if eye movements predict turn direction independently of whisker movements. Have you considered using a model that includes both variables, and/or performing a partial correlation analysis?

We performed additional analysis to examine the unique contributions of whisker asymmetry and eye positions in predicting the turn direction (Figure 3C,D). We found no significant additional effects of shuffling either variable alone, indicating that information of the two variables largely overlap with each other. We added the results as Figure 3CD, and revised the manuscript (lines 258-275, 361-377, 683-701).

-Discussion, first paragraph: “to recognize an adjacent dark lane”. What do you mean?

We have rewritten this.

-Discussion is missing a limitations section to discuss the limitations of your work, including - head fixing and sample sizes.

We have added a discussion about the limitations of this work (lines 486-500).
